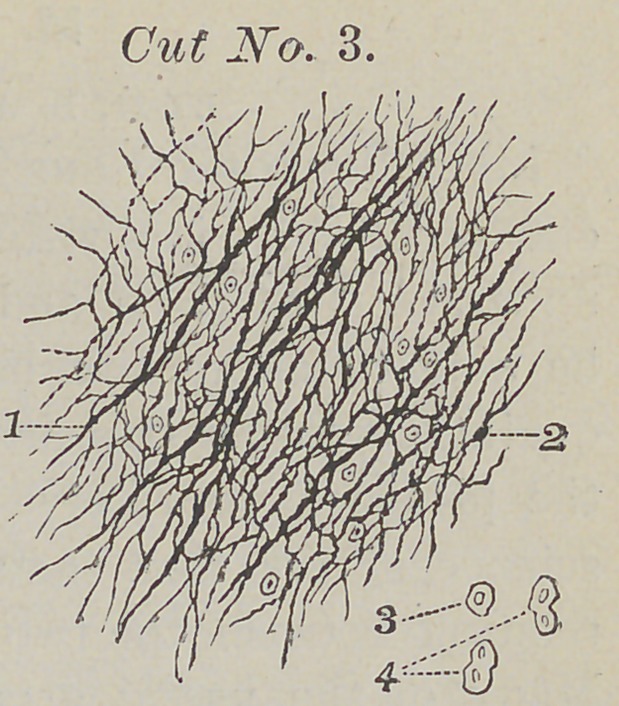# The Structure and Development of the Teeth

**Published:** 1872-07

**Authors:** Geo. B. Harriman

**Affiliations:** Boston, Mass.


### Dental Register.

VOL. XXVI.] JULY, 1872. No. 6.]

### COMMUNICATIONS.

### The Structure and Development of the Teeth.

PROF. GEO. B. HARRIMAN, BOSTON, MASS.

The period has passed for success to the dentist who relies only upon his knowledge of mechanism and his dexterous use of mechanical implements. Since dentistry became a specialty, a distinct profession, if the practitioner could extract a tooth and possibly insert a new one to supply the place of one decayed and lost, his accomplishment was successful in satisfying the public and in securing a lucrative practice. At the present period this can not be done.

Now a thorough knowledge of the human system, histological, physiological, and anatomical, and a rigid course of study at a dental college is demanded, before a diploma can be granted or a practitioner have any encouragement or success. It is as preposterous to think of entering the dental profession at the present day without being master of the elementary branches specified above and taught in our dental colleges, as to think of entering the medical, or legal, or theological without a long and thorough training in the institutions where instruction is given in the branches which underlie these professions, and a knowledge of which is indispensable to honorable graduation and acknowledgment of ability by those appointed to certify and practice in the same.

Scientific attainments must be supplemented by skill in mechanism, enabling the dentist to produce an indvidual tooth or a set which will truly supply nature's loss both in symmetry and anatomical adaptation.

After laying this foundation it is not to be considered that all labor is completed and need of further toil overcome. Science is not stationary, but ever developing, and hence investigation must be continuous, and proving what is thus developed by practice, a constant labor.

Nor will it subserve the best interests of the dentist who is looking for honor in being profound, or wealth in being expert and skillful, to trust to others for investigation and proof. He must be ever watchful to discern the new dawnings of science, and ever interested in inquiring how such developments can be used in advancing the interests of dentistry. To be thus accomplished his library must be stocked with the best authors, and his laboratory be supplied with all necessary and convenient appointments. In the one he should be a most diligent student, and in the latter a most active and expert worker. Without great interest in the profession, and strong desire for the greatest success and eminence therein, together with constant resolution to neglect no oportunity for improvement, both the library will be either meagre and unfrequented, and the laboratory be destitute or not much used but for current work. In either case the dentist will be but little known, and his practice be inefficient and imperfect.

Having thus treated of what enters into the preparation of the dentist for his profession, and briefly considered what is necessary to give him distinction and success, I now come to the specific object of the present article, namely, the *constituents of the human teeth.*

A firm bed for these teeth has been laid by the great Artificer of the human body in the jaws of the mouth, or in the alveolar processes of the superior and inferior maxillary bones. In other departments of the animal economy where teeth exist their position varies from the mouth to the pylorus.

In the human body the number of temporary or deciduous teeth is twenty, and the number of permanent teeth, thirty-two.

In many of the herbivorous animals, that class which chew the cud, the number of teeth is the same as in the human mouth, though somewhat differently arranged.

The ox, sheep, and antelope have no superior, temporary or permanent incisors, nor canine teeth.

The number of deciduous teeth is twenty, six inferior incisors, two inferior canines, six inferior molars and six superior molars.

The arrangement of the permanent teeth is as follows:

| | | | |
|---|---|---|---|
| Superor canines | 0—0. | Superior incisors | 0—0. |
| Inferior canines | 1—1. | Inferior incisors | 3—3. |
| Superior molars | 3—3. | Superior premolars | 3—3. |
| Inferior molars | 3—3. | Inferior premolars | 3—3. |
| Total | | | 32. |

The formation and growth of the teeth in this order of animals is exceedingly rapid and interesting to the investigator. Careful and persistent study of their structure and development will result in valuable information which can be turned to good account by the dentist.

In many of the vertebrate animals are found thirty-four teeth. A variation, however, is here discovered of from one tooth to one hundred and forty. Not only is there a difference discernable in numbers and position, but in form, body, and microscopical structure, and in the versatile relations which the diversified tissues entering their composition sustain to each other.

Whilst studying this difference it is important to consider the habits and food of these animals; for the teeth, it must be remembered, are the primary agents in compounding the diet, and reducing it by mastication for the indispensable changes to which it is subjected by the residue of the nutritive functions. In the alveolar process of the superior and inferior maxillary bones in man is the matrix, over which are several layers of epithelium, and the mucous membrane, thus imparting to the matrix the power of producing teeth. The constituency of the teeth is enamel, dentine and cementum.

The periosteum, which is composed of cells and fibres of connective tissue, is the vital portion of the cementum (Crustapetrosa), and carries a solution of lime salts which are essential to the construction of the cementum. Here we discover what is termed by Harris "Exostosis Dentinum," and by J. H. Mc.Quillen, "Hypertrophy of the Cement." This latter term is singularly inappropriate and absurd. Hypertrophy designates those conditions where the individual elements of a structure take up a considerable amount of different matter, and thereby become larger, and in consequence of the simultaneous enlargement of the number of elements, at last the entire organ becomes swollen. Prof. McQuillen uses this term in describing histological or pathological *new* formations of the cementum, which the least learned in the profession at once pronounce decidedly out of place. However, this writer is so noted in his misapplication of terms and in the use of meaningless words and phrases, that where he is known he will not be likely to lead astray. Hypertrophy of the liver may be spoken of when it becomes enlarged by the cells taking up more than the necessary supply of substance, and not a development of a series of small cells, for that would be a hyperplastic condition of the organ; the muscle becomes thicker, all its primitive fasciculi become swollen, and the skin likewise enlarges by reason of single fat cells absorbing more than their usual quantity of fat. The cementum does multiply its cells, but does not swell.

Exostosis is produced by the deposit of calcareous salts by the fibres of the periosteum. Though exostosis (an objectionable word) and cementum have different names, yet under the microscope it is impossible to detect any difference. The word hyperplasia of cementum would better express the character of the structure than either hypertrophy or exostosis. The hyperplastic process in all cases produces a tissue similar to that of the original part, and this proves to be the fact on examination of the enlargement of the cementum.

In the development of the tooth the enamel first receives the lime salts. The enamel cells and fibres become nearly calcified anterior to the formation of the dentine. A pulp passes through the center of each tooth which contains an artery, veins, a great number of cells and fibres of connective tissue, and an immense number of small nerve fibres.

Though laid down in works on dentistry and generally acknowledged by the profession as correct, I am persuaded, after careful examination and frequent and varied experiments, there is no such thing as enamel pulp. The pulp above mentioned as passing through the center of the tooth is a jelly-like mass of connective tissue, nerve fibres, and blood vessels, which when calcified leave traces of fibres similiar to those in dentine.

*Cut No.* 1.

Figure one in this cut points to the uncalcified fibres; figure two points to a small portion of tissue; figure three is intended to represent the calcareous salts.

The enamel, prior to the deposit of any calcareous substance, is much like this pulp, wherein are very fine cells and fibres which can be detected with a medium high power mioscopie, magnifyng from seven to ten hundred times. These fibres, when artistically prepared for microscopic examination, show that they are not more than one twenty-thousandth of an inch in diameter. They are supplied with calcareous salts by the dental artery, until nearly entire calcification obtains, and on becoming thus calcified they lose the name of fibres and acquire that of enamel, or in other phraseology the enamel is calcified fibres. When fully formed the enamel becomes smooth and glassy, and is the hardest of all the substances belonging to the human body.

*Cut No. 2.*

Figure one in cut number two, points to that part of the enamel which is nearly formed. Figure two shows portions of soft-solid tissue Figure three designates a small portion of calcareous salts.

Within and beneath the enamel is a jelly-like gelatinous body of fibres, and small, young nucleated cells, from which the dentine is formed, which are easily discerned. In a few instances I have detected *two nuclei* with a *division of the cell* taking place, showing distinctly a proliferation in this particular tissue (see cut number three, figures three and four), previous to the deposit of the calcareous salts. The tissues forming the dentine are one homogenous mass of fibres and small young cells, measuring, when carefully detached with fine pointed needles, not more than the eighteen-thousandth of an inch in diameter. The cells and fibers absorb the solution of lime salts received from the dental artery. They take up like a sponge or blotting paper. It hence appears that the dentine is constructed from and by the deposit of lime salts interstertially around the cells and fibres. As they continue to form and take up the solution of lime salts, the tooth is constantly pressing toward the mucous membrane until the eruption and entire formation of the tooth. If we take one of these gelatinous bodies and allow it to harden in diluted chromic acid, and then make a very thin section with some sharp instrument, by the addition of the terchloride of gold with the aid of an one-fiftieth immersion objective, countless fine, pale fibres can be seen, measuring not more than the fifty-thousandth of an inch in diameter.

Cut number three is magnified about forty-five diameters. Figure one in this cut designates the nerve fibres. Figure two points to the nerve ganglion or cell. Figure three shows the nucleated cell. Figure four designates a cell with two nuclei with a division taking place in the cells.

The existence of dentinal fibres or nerve fibres in the dentine is disallowed by Prof. McQuillen, who regards them as a *post mortem* result, proceeding from the extraction of the

*Cut No. 3.*

tooth, and "due to the coagulation of fibrin of the liquor sanguinis rather than a normal condition of a living organ." Such a position shows the stupdity which overcomes the mind in an attempt to convert truth into error. Should the brinfi coagulate, which Prof. McQuillen alleges to exist in the dentine, the amount would be so exceedingly small as to escape detection except by the highest magnifying powers. The analysis of the blood demonstrates that there is but about two per cent. of fibrin in blood, and all physiologists agree that fibrin will not coagulate when apart from the red blood corpuscle.

Prof. McQuillen intimates his liability to mistake, and he may here retract, as he has been compelled to do in other cases, and thus reluctantly show that others as well as himself can impart knowledge.

Not only in these remarks on the normal or formal structure of the teeth am I in conflct with Prof. McQuillen, but with other writers in the advocacy of theories yet held in great consideration as the correct histology by men of high standing in our profession. These other writers, however, do not ordinarily stultify themselves so completely as we have shown to be the case with Prof. McQuillen. I am aware my positions may provoke animadversion and criticism, but I do not fear confutation, knowing that by long, careful, and laborious investigations I have reached my conclusions.

In another article I shall treat of dental development.

## Figures and Tables

**Cut No. 1. f1:**
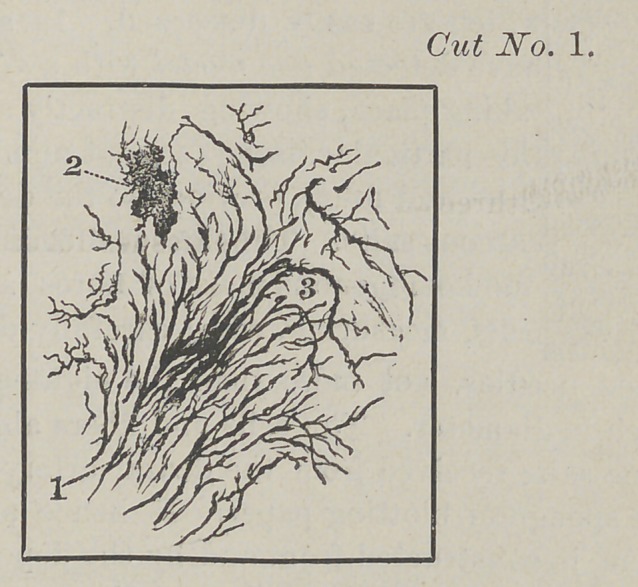


**Cut No. 2. f2:**
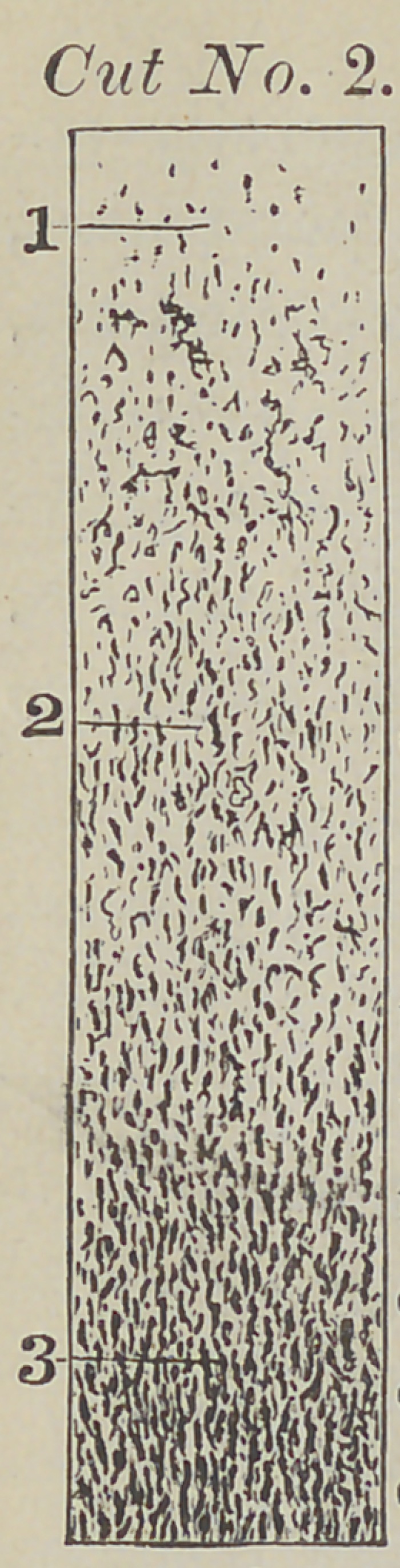


**Cut No. 3. f3:**