# Erratum

**Published:** 1872-08

**Authors:** 


### Erratum.

In July No. page 282, eighth line from bottom, read forty-five hundred instead of forty-five. Also on page 283, seventh line from the top, read fibrin instead of brinfi.

